# Maize-grain legume intercropping for enhanced resource use efficiency and crop productivity in the Guinea savanna of northern Ghana

**DOI:** 10.1016/j.fcr.2017.07.008

**Published:** 2017-11

**Authors:** Michael Kermah, Angelinus C. Franke, Samuel Adjei-Nsiah, Benjamin D.K. Ahiabor, Robert C. Abaidoo, Ken E. Giller

**Affiliations:** a Plant Production Systems, Wageningen University, P.O. Box 430, 6700 AK Wageningen, The Netherlands; b Soil, Crop and Climate Sciences, University of the Free State, P.O. Box 339, Bloemfontein 9300, South Africa; c International Institute of Tropical Agriculture, P.O. Box TL 06, Tamale, Ghana; d CSIR-Savanna Agricultural Research Institute, P.O. Box 52, Tamale, Ghana; e Department of Theoretical and Applied Biology, Kwame Nkrumah University of Science and Technology, PMB, Kumasi, Ghana

**Keywords:** Soil fertility, Spatial arrangement, Radiation interception, LER, Net benefit

## Abstract

Smallholder farmers in the Guinea savanna practise cereal-legume intercropping to mitigate risks of crop failure in mono-cropping. The productivity of cereal-legume intercrops could be influenced by the spatial arrangement of the intercrops and the soil fertility status. Knowledge on the effect of soil fertility status on intercrop productivity is generally lacking in the Guinea savanna despite the wide variability in soil fertility status in farmers’ fields, and the productivity of within-row spatial arrangement of intercrops relative to the distinct-row systems under on-farm conditions has not been studied in the region. We studied effects of maize-legume spatial intercropping patterns and soil fertility status on resource use efficiency, crop productivity and economic profitability under on-farm conditions in the Guinea savanna. Treatments consisted of maize-legume intercropped within-row, 1 row of maize alternated with one row of legume, 2 rows of maize alternated with 2 rows of legume, a sole maize crop and a sole legume crop. These were assessed in the southern Guinea savanna (SGS) and the northern Guinea savanna (NGS) of northern Ghana for two seasons using three fields differing in soil fertility in each agro-ecological zone. Each treatment received 25 kg P and 30 kg K ha^−1^ at sowing, while maize received 25 kg (intercrop) or 50 kg (sole) N ha^−1^ at 3 and 6 weeks after sowing. The experiment was conducted in a randomised complete block design with each block of treatments replicated four times per fertility level at each site. Better soil conditions and rainfall in the SGS resulted in 48, 38 and 9% more maize, soybean and groundnut grain yield, respectively produced than in the NGS, while 11% more cowpea grain yield was produced in the NGS. Sole crops of maize and legumes produced significantly more grain yield per unit area than the respective intercrops of maize and legumes. Land equivalent ratios (LERs) of all intercrop patterns were greater than unity indicating more efficient and productive use of environmental resources by intercrops. Sole legumes intercepted more radiation than sole maize, while the interception by intercrops was in between that of sole legumes and sole maize. The intercrop however converted the intercepted radiation more efficiently into grain yield than the sole crops. Economic returns were greater for intercrops than for either sole crop. The within-row intercrop pattern was the most productive and lucrative system. Larger grain yields in the SGS and in fertile fields led to greater economic returns. However, intercropping systems in poorly fertile fields and in the NGS recorded greater LERs (1.16–1.81) compared with fertile fields (1.07–1.54) and with the SGS. This suggests that intercropping is more beneficial in less fertile fields and in more marginal environments such as the NGS. Cowpea and groundnut performed better than soybean when intercropped with maize, though the larger absolute grain yields of soybean resulted in larger net benefits.

## Highlights

• Productivity of different intercropping patterns was tested in Guinea savanna of northern Ghana.
• Land Equivalent Ratios in intercropping systems are greater under low soil fertility conditions.
• Competitive balance between intercrops in poor fields leads to greater Land Equivalent Ratios.
• Within-row maize-legume intercropping is more productive than distinct row systems.
• Radiation use efficiency is higher in intercrops than in sole crops.

## 1 Introduction

The Guinea savanna of West Africa is characterised by poor and declining soil fertility due to continuous cereal-based cropping systems without adequate soil nutrient replenishment (Dakora et al., 1987, Sanginga, 2003). The declining soil fertility coupled with an erratic unimodal rainfall regime has increased the risk of crop failure in sole cropping systems. Intercropping, the simultaneous or sequential growing of two or more crop species on the same piece of land (Willey, 1990), could mitigate risk of crop failure. For instance, in case the main crop (typically maize, *Zea mays* L.) fails to produce yield due to erratic distribution of rainfall within a season, the added grain legume provides food for the farm household (Rusinamhodzi et al., 2012). Consequently, farmers in the Guinea savanna commonly practise cereal-legume intercropping to safeguard household food and income. The inclusion of grain legumes is essential for soil fertility sustenance as they contribute to soil fertility enhancement through biological fixation of atmospheric nitrogen (N_2_) and N mineralised from legume residues (Giller, 2001). Legumes also provide grain rich in protein and minerals for household nutrition and income (Giller, 2001).

The greater crop yields and productivity of intercrops relative to sole crops result from complementary use of resources for growth by the intercrop components (Willey, 1979, Ofori and Stern, 1987, Rao and Singh, 1990, Willey, 1990). Differences in acquisition and use of light, water and nutrients by the different intercrop components (Ofori and Stern, 1987, Willey, 1990) results in inter-species competition being smaller than intra-species competition (Vandermeer, 1989). The complementary effect can be temporal where peak demands for resources by component crops occur at different times or spatial where complementary resource use occurs due to differences in canopy and root structures (Willey, 1990). Complementarity is also likely as intercropped maize uses N from the soil for growth whilst the legume can rely more on atmospheric N_2_-fixation for growth. These can be influenced by soil fertility status, spatial planting arrangements and choice of intercrop components (Midmore, 1993). Weeds and diseases may be better suppressed in intercropping than in sole cropping although this may be influenced by the intercropping pattern and the resulting canopy structure (Liebman and Dyck, 1993, Trenbath, 1993).

Spatial intercropping patterns have been studied in the Guinea savanna of northern Ghana (e.g. Agyare et al., 2006; Konlan et al., 2013) and Nigeria (e.g. Ajeigbe et al., 2010) mainly under controlled conditions. All these studies assessed the performance of different distinct alternate row intercropping patterns of maize and legumes. Rusinamhodzi et al. (2012) reported greater LER when the intercrops were planted in the same row rather than in distinct rows in Central Mozambique. Other studies (Agyare et al., 2006; Konlan et al., 2013) generally showed intercrop advantages over sole crops that declined as the width of adjacent strips of each crop was increased. For instance, Konlan et al. (2013) reported a larger LER for 1:1 alternate rows of maize and groundnut than for 2:2 alternate row intercrops. In some cases, sole crops were more productive than intercrops when two or more rows of intercropped maize were alternated with the same number of groundnut (*Arachis hypogaea* L.) rows (Konlan et al., 2013).

Knowledge on the ecological and economic performance of within-row maize-legume intercrop pattern in relation to the distinct row intercrop patterns and sole crops is limited to controlled trials in the Guinea savanna region. Studies conducted in Turrialba, Costa Rica (Chang and Shibles, 1985) and Western Australia (Ofori and Stern, 1986) reported greater maize-cowpea (*Vigna unguiculata* (L.) Walp) intercrop advantages under low soil N and P conditions. Searle et al. (1981) and Ahmed and Rao (1982) also observed larger maize-soybean (*Glycine max* (L.) Merr.) intercrop advantages when soil N fertility was poor. As smallholder farms in the Guinea savanna vary widely in soil fertility status, a better understanding of the relative performance of intercrop in relation to soil fertility is required. We studied the effects of soil fertility status and different spatial maize-legume intercropping patterns and monocultures on grain yields, intercrop efficiency and productivity and economic profitability in contrasting sites in the southern and northern Guinea savanna agro-ecological zones of northern Ghana.

## 2 Materials and methods

### 2.1 Study sites and on-farm experiments

The trials were conducted on farmers’ fields in the cropping seasons of 2013 and 2014. The sites were Kpataribogu (9°58′ N, 0°40′ W) in Karaga District (southern Guinea savanna, SGS; 1076 mm mean annual rainfall) and Bundunia (10°51′ N, 1°04′ W) in Kassena-Nankana East Municipal (northern Guinea savanna, NGS; 990 mm mean annual rainfall) in northern Ghana. Both sites have a single rainy season which extends from May to October in SGS and from June to October in NGS. The soils at both sites are predominantly sandy soils classified as Savanna Ochrosol and Groundwater Laterites in the Interim Ghana Soil Classification System (Adjei-Gyapong and Asiamah, 2002) and as Plinthosols in the World Reference Base for soil resources (WRB, 2015).

At each site, three field types representing a highly fertile field (HF), a medium fertile field (MF) and a field low in fertility (LF) were selected and used for both seasons. Fields were selected using farmers’ knowledge with the assistance of Agricultural Extension Officers, followed by soil physico-chemical analysis. The selected fields were under mono-cropping in the three preceding seasons, *i.e.* in the SGS site HF: soybean-groundnut-maize, MF: maize-soybean-maize, LF: groundnut-soybean-cotton; in the NGS site HF: maize-maize-maize, MF: maize-groundnut-fallow, LF: maize-maize-groundnut. Previously mono-cropped fields were selected to reduce within-field variability. Soils were sampled at 0–15 cm depth at each trial field prior to land preparation in 2013. All soil cores were thoroughly mixed and about 1 kg sub-samples per field were air-dried and passed through a 2 mm-mesh sieve. These were analysed for pH (1:2.5 soil:water suspension), organic C (Walkley and Black), total N (Kjeldahl), available P (Olsen), exchangeable K, Mg, and Ca (in 1 M ammonium acetate extracts) and texture (hydrometer method). Some of these soil physico-chemical analysis data presented in Table 2 are reported in Kermah et al. (submitted).

### 2.2 Experimental design, treatments and crop management

Three grain legumes cowpea (CP), soybean (SB) and groundnut (GN) were intercropped with maize (MZ) in different spatial arrangements: (i) maize-legume intercropped within-row, (ii) one row of maize alternated with one row of legume, (iii) two rows of maize alternated with two rows of legume, (iv) a sole crop of maize and (v) a sole crop of legume. For the within-row treatments, a maize planting hill alternated two equally spaced cowpea or groundnut hills, or four soybean hills within the same row. An inter-row spacing of 75 cm was maintained for all treatments and crops. Intra-row spacing was 50 cm for intercropped maize within-row, 25 cm for sole maize and all distinct rows intercropped maize and for sole cowpea and sole groundnut. Soybean had an intra-row spacing of 12.5 cm in both the distinct row intercrops and the sole crop. Maize (intercropped and sole) and all legumes within-row treatments were sown at one seed per hill, while all distinct row and sole legume treatments were sown at two seeds per hill. The resultant plant sowing densities (plants ha^−1^), respectively for intercrops and sole crops were: maize (26,667 and 53,333), cowpea and groundnut (53,333 and 106,666) and soybean (106,666 and 213,332). The experiment was conducted in a randomised complete block design with blocks of treatments replicated four times per fertility level at each site. Treatments were randomised within blocks and a plot measured 4.5 m × 4.0 m.

The land was ploughed with a tractor and ridged manually in the SGS and with a tractor in the NGS, reflecting the common practices at both sites. Sowing was done on the top of the ridges using locally preferred crop varieties: cowpea–Padi-tuya (SARC 3–122-2); soybean–Jenguma (Tgx 1448-2E); groundnut–Chinese and maize–Obatanpa (GH83-63SR). Groundnut variety, Samnut 22 was used in 2013 in the SGS. In 2013, all crops were sown simultaneously (July 1–2 in the SGS; July 16–17 in the NGS) due to the late onset of rains. Sowing in 2014 followed the recommended sowing times: maize-groundnut on June 13, maize-soybean on July 4 and maize-cowpea on July 17 in the SGS. All crops in the NGS were sown on July 15 due to the late onset of rains in 2014. Cowpea was sprayed twice at flowering and podding stages with lambda-cyhalothrin (in the SGS) and cypadem 43.6 EC (36 g cypamethrin and 400 g dimethoate per litre) (in the NGS) in the form of an emulsifiable concentrate at a rate of 0.75-1.00 l ha^−1^ for sole cowpea and 50% of that dosage for intercropped cowpea for each insecticide depending on the presence and population of pests (flower thrips: e.g. *Megalurothrips sjostedti* Tryb. and pod borers: e.g. *Maruca vitrata* Fab.). Soybean seeds were inoculated with Legumefix (LegumeTechnology, UK) *Bradyrhizobium japonicum* strain 532c (re-isolated in Brazil from strain USDA 442 Wisconsin, USA) at a rate of 5 g inoculant per kg seed. All treatments received uniform applications of 25 kg P ha^−1^ as TSP and 30 kg K ha^−1^ as muriate of potash at sowing. Nitrogen in the form of urea was spot-applied to maize at 25 kg N ha^−1^ for intercrops and 50 kg N ha^−1^ for sole crops in two equal split doses at three and six weeks after sowing (WAS). All fertilisers were placed 5 cm from the plants at 3 cm depth. All fields were weeded twice with a hoe at 3 and 6 WAS.

### 2.3 Field measurements

Daily rainfall during the season was measured with rain gauges installed at each site. Photosynthetically active radiation (PAR) interception was measured with AccuPAR LP-80 Ceptometer (Decagon Devices Inc., Pullman, Washington). Measurements were made above and below the crop canopies in each plot at four randomly selected locations. Five successive PAR readings each above and below the canopy were taken and averaged per location with the Ceptometer placed across the crop rows. PAR measurements were made generally under clear skies between 10.00 and 14.00 h, at 10-15 days’ intervals (depending on weather conditions). In the within-row intercrop plots, PAR was measured by considering the whole canopy of the legume and maize components. In the 1:1 and 2:2 distinct row intercrop plots, PAR readings were taken separately across legume and maize rows and averaged.

Legume biomass was sampled at the mid-pod filling stage from an area of 3.0 m × 1.0 m by cutting plants at the soil surface, separated into shoots and pods, and both total and sub-sample fresh weights taken in the field. Legume and maize grain yields were measured just after physiological maturity by harvesting a 3.0 m × 1.5 m area excluding the border rows. Maize ears and stalks were harvested and the sheaths were removed by hand. Fresh weights of all cobs and of sub-samples of ten randomly picked cobs were determined in the field. Total and sub-sample fresh weights of legume pods were taken in the field. Conversion factors for the different plant parts were derived from experimental data from trials conducted in the Guinea savanna of northern Ghana and Nigeria. Pooled means of the various treatments were taken and used to calculate the dry weights of the sub-samples (values given are dry matter fractions): Cowpea (mid-pod stage: shoot = 0.17, pod = 0.18; crop maturity: pod = 0.64, grain to pod ratio = 0.77), soybean (mid-pod stage: shoot = 0.29, pod = 0.31; crop maturity: pod = 0.69, grain to pod ratio = 0.71), groundnut (mid-pod stage: shoot = 0.22, pod = 0.31; crop maturity: pod = 0.66, grain to pod ratio = 0.64) and maize (crop maturity only: cob = 0.71, grain to cob ratio = 0.79). These conversion factors are reported in Kermah et al. (submitted). Grain yields are presented at 14% moisture for maize and 12% moisture for legumes; above-ground dry matter yields on dry weight basis.

### 2.4 Assessment of intercrop productivity and profitability

The Land Equivalent Ratio (LER) was used to evaluate resource use efficiency and the productivity of intercrops. LER values above one indicate that intercropping is more productive and efficient in using environmental resources than sole cropping, and values less than one that sole crops were more productive. Individual within-block values of maize or legume grain yields were used as the denominator values to calculate LER.

(1) *LER* *=* *Y_il_/Y_sl_* *+* *Y_im_/Y_sm_*

where *Y_il_* and *Y_im_* are intercrop yields of legume and maize respectively while *Y_sl_* and *Y_sm_* are the sole yields of legume and maize (Mead and Willey, 1980).

A partial budget analysis, accounting of the total variable costs and gross returns of a production system to determine a change (increase or decrease) in profit (Alimi and Manyong, 2000) was done. Net benefit used to determine the relative economic profitability of the cropping systems.

(2) Net benefit = Total revenue (TR) − Total cost (TC)

Total revenue was estimated as the product of grain yield (t ha^−1^) and grain price (US$ t^−1^). Grain prices were obtained from local market surveys at harvest time when most farmers sell their produce. TC was the sum of the costs of input (seeds, fertilisers and agro-chemical) and labour for the different field activities. Labour cost for each activity was based on the local daily wage per person to perform the activity ha^−1^ and multiplied by the total man-days required to complete the activity under sole maize and legume conditions. TC of the intercrop pattern was the sum of 50% of the TC of each sole crop. For the within row intercrops, the costs of sowing, urea application to maize and weeding were calculated as 68% that of the respective sole crops. This was based on the assumption that those activities require 18% more labour in an intercrop (Rusinamhodzi et al., 2012). Details of unit costs, grain prices and estimated labour requirements are presented in Table 1a, Table 1b. Net benefits were estimated for each season and averaged.

**Table 1a:** Unit input and labour costs and grain prices used in estimating total cost (TC) and total revenue (TR) in the southern Guinea savanna (SGS) and northern Guinea savanna (NGS) of northern Ghana.

	SGS		NGS	
	2013	2014	2013	2014
Input costs (US$ ha^−1^)				
Maize seeds	9.0	6.6	7.6	7.6
Soybean seeds	40.0	27.0	39.5	28.6
Groundnut seeds	56.2	37.7	59.6	47.4
Cowpea seeds	37.5	20.1	30.4	25.2
Urea	54.3	50.4	54.3	50.4
TSP	99.5	66.0	99.5	66.0
MoP	51.1	33.9	51.1	33.9
Insecticide	6.5	4.0	6.5	4.0
Inoculant	15.0	15.0	15.0	15.0

Labour input (US$ ha^−1^)				
Ploughing	43.2	32.7	74.0	57.3
Ridging	74.0	49.1	61.7	49.1
Sowing	6.8	4.9	8.6	4.9
Fertiliser application	6.2	4.9	6.2	4.9
Spraying	6.2	4.9	8.6	4.9
Weeding	8.6	6.6	8.6	6.6
Harvesting	8.6	6.6	8.6	6.6
Threshing	4.9	4.1	4.9	4.1

Grain prices (US$ kg^−1^)				
Maize	0.51	0.38	0.37	0.36
Soybean	0.88	0.76	0.95	0.67
Groundnut (shelled)	1.86	1.43	2.52	1.79
Cowpea	1.12	0.76	1.17	0.95

Exchange rate for costs: GH¢2.00 = US$1.00 in 2013; GH¢3.02 = US$1.00 in 2014 (average rate for each year, i.e. inputs acquisition to harvest). Exchange rate for grain prices: GH¢2.08 = US$1.00 in 2013; GH¢3.20 = US$1.00 in 2014 (average rate for 3rd and 4th quarters of each year, i.e. harvest and selling period). Exchange rates were obtained from Bank of Ghana quarterly bulletin.

**Table 1b:** Estimated labour requirements (days ha^−1^) of field operations of maize and legumes under sole crop systems used in estimating TC.

Activity	Cowpea	Soybean	Groundnut	Maize	Source
Sowing	12	17	11	10	Franke et al. (2010)
P&K application	2	4	2	2	Ojiem et al. (2014)
N application	–	–	–	7	Franke et al. (2006)
Spraying	2	–	–	–	Own observation
First weeding	36	36	36	25	Franke et al. (2006)
Second weeding	30	30	30	21	83% of first weeding^a^
Harvesting	14	14	34	12	Franke et al. (2010)
Threshing	17^b^	29	46^c^	23	Franke et al. (2006)

^a^Heemst et al. (1981).

^b,c^Ojiem et al. (2014).

^b^Includes the shelling of groundnut.

**Table 2:** Physical and chemical properties of the three types of fields differing in soil fertility in the southern Guinea savanna (SGS) and northern Guinea savanna (NGS) agro-ecologies of northern Ghana. The SED represents the standard error of difference between means.

	SGS				NGS			
Soil fertility parameter	HF	MF	LF	SED^a^	HF	MF	LF	SED^a^
pH	6.2	5.4	5.8	0.3	5.4	4.3	4.7	0.5
Organic C (g kg^−1^)	10.9	9.0	7.4	1.4	6.2	3.1	3.9	1.3
Total N (g kg^−1^)	0.9	0.8	0.8	0.05	0.6	0.3	0.2	0.2
Olsen P (mg kg^−1^)	2.6	2.6	1.7	0.4	2.8	2.6	1.9	0.4
K (cmol_+_ kg^−1^)	0.3	0.2	0.2	0.05	0.2	0.1	0.1	0.05
Ca (cmol_+_ kg^−1^)	1.7	1.6	1.3	0.2	1.6	0.5	0.8	0.5
Mg (cmol_+_ kg^−1^)	0.7	0.6	0.7	0.05	0.9	0.1	0.7	0.3
ECEC (cmol_+_ kg^−1^)	10.2	6.6	5.2	2.1	6.9	1.8	3	2.2
Sand (g kg^−1^)	563	738	538	89	738	883	798	59
Silt (g kg^−1^)	321	180	400	91	160	101	160	28
Clay (g kg^−1^)	116	81	61	23	101	16	41	36

a SED represents the standard error of differences between means and was calculated following the procedure described by Saville (2003).

### 2.5 Data handling and analysis

The percentage intercepted PAR (% IPAR) was calculated following Gallo and Daughtry (1986) as:

(3) *%IPAR* = [1–(*It/Io*)] × 100

where *It* is the PAR measured just below the lowest green leaves (lowest layer of photosynthetically active leaves) while *Io* is the incident PAR.

The expected intercepted PAR (IPAR) by intercrops based on plant densities was calculated as: (0.5 × sole maize IPAR) + (0.5 × sole legume IPAR). The expected IPAR needed by intercrops to produce the observed combined intercrop grain yields if RUE is similar to that of sole crops was calculated as: {(Sole maize IPAR × (intercrop maize grain yield/Sole maize grain yield)} + {(Sole legume IPAR × (intercrop legume grain yield/Sole legume grain yield)}.

Statistical analysis was conducted using GenStat (version 18.1, VSN International Ltd). The different maize-legume systems and sites were analysed separately initially, and then combined. Data were analysed with a linear mixed model with planting arrangement, soil fertility status and site (for cross site analysis) as fixed factors and replication as random factor to test for effect of planting arrangement, soil fertility and site on crop yields and intercrop productivity (assessed with LER). Analysis of covariance (ANCOVA) to explain the sources of variation in above-ground dry matter and grain yields as well as land equivalent ratios were conducted using the general ANOVA structure with planting arrangement as a fixed factor, replication as random factor and measured total soil N and available P as covariates. For PAR interception, repeated measurements analysis was done with plots as subjects and measurement dates (presented as days after sowing, DAS) as time points. Measurement date × cropping system × soil fertility were kept as fixed factors with the models fitted for correlation within subjects across time using antedependence model order 1 since the intervals between different measurement dates were not equally spaced. The standard error of differences between means (SED) was used to compare treatment means at *P <* 0.05 significance level.

## 3 Results

### 3.1 Soil fertility and rainfall distribution

**Fig. 1:**
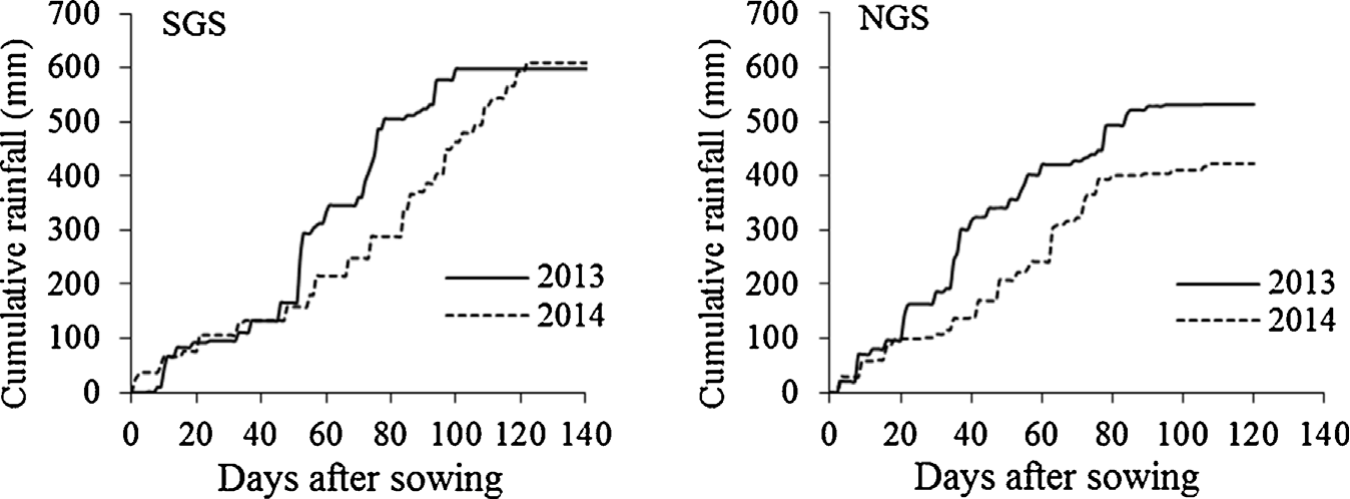
Cumulative rainfall during the 2013 and 2014 growing seasons. In 2014, 0 DAS in the southern Guinea savanna (SGS) refers to the sowing date of the maize-groundnut system (June 13). Maize-soybean and maize-cowpea systems were sown 21 and 34 days later, respectively.

The site in the SGS received more rainfall than the NGS in both seasons (Fig. 1). Total rainfall during the growing season was 598 mm in 2013 and 609 mm in 2014 in the SGS, and 532 mm in 2013 and 423 mm in 2014 in the NGS. The rainfall at both sites was below the long term mean seasonal rainfall values: 861 mm for the SGS and 807 mm for the NGS (Ghana Meteorological Agency, Legon, Accra).

The SGS had relatively more fertile soils with values for pH, OC, N, exchangeable cations and clay content more favourable for crop growth than the NGS. Available P and exchangeable K were low at both sites. The relatively sandy soils in the NGS were likely to have a low moisture holding ability, while the low soil pH could reduce the availability of micronutrients. Soil OC was sub-optimal for good soil nutrient retention and soil N supply, and likely to limit crop growth at both sites. Exchangeable Ca and Mg were unlikely to limit crop growth at both sites.

Soil chemical analysis largely confirmed the soil fertility classification by the farmers. In the SGS, pH, OC, ECEC and clay content were more favourable for crop growth in the HF field than in the MF field, while both fields had generally larger values of OC, P, exchangeable Ca and ECEC than the LF field (Table 2). In the NGS, the HF field had soil fertility characteristics more favourable for crop growth compared with the MF and LF fields, while the latter two were comparable in most cases (Table 2).

### 3.2 Radiation interception, above ground biomass and grain yields

**Fig. 2:**
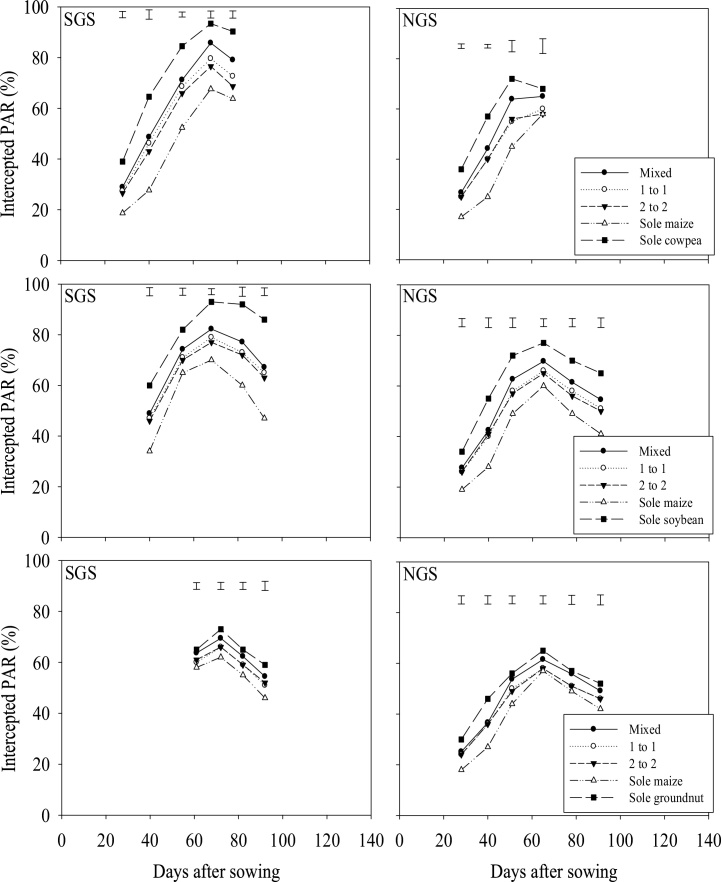
Percentage intercepted PAR as affected by cropping pattern in 2014, averaged over soil fertility levels in the SGS and the NGS of northern Ghana. The error bars indicate the combined standard error of differences between means (SED) for cropping patterns.

**Fig. 3:**
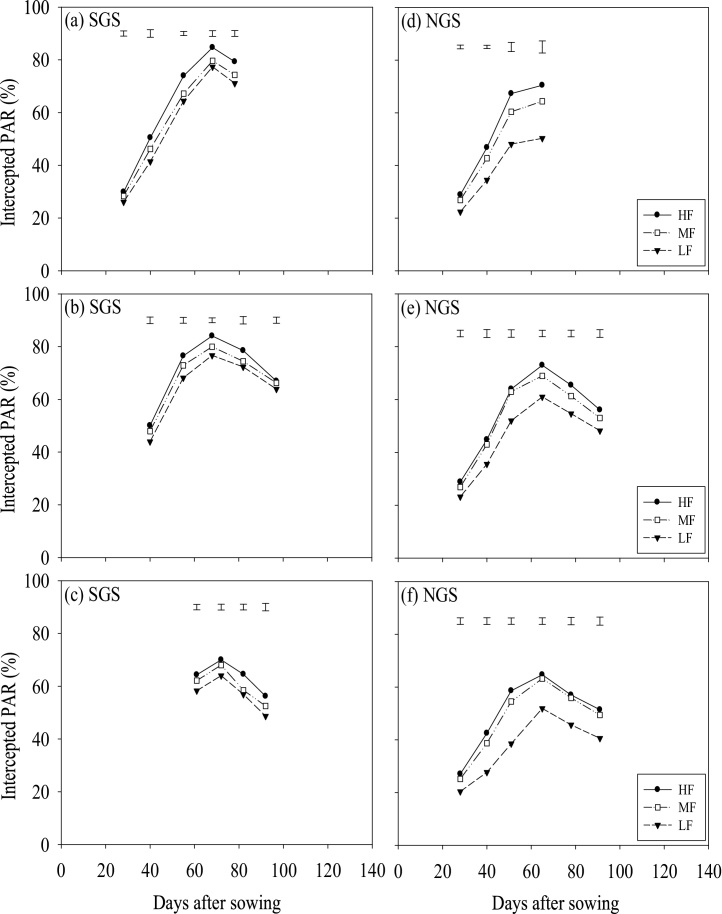
Percentage intercepted PAR as affected by soil fertility status in 2014 in (a) maize-cowpea, (c) maize-soybean and (e) maize-groundnut systems in the SGS and in (b) maize-cowpea, (d) maize-soybean and (f) maize-groundnut systems in the NGS of northern Ghana. Data are averaged over cropping systems. Error bars indicate the combined standard error of differences between means (SED).

Sole legumes intercepted more PAR than intercrops (*P <* 0.001; Fig. 2) whilst intercrops intercepted more PAR than sole maize. This was more evident after silking when maize leaves started senescing. Differences in intercepted PAR between intercrop patterns were not significant at the initial growth stages until flowering of legumes and maize. Thereafter, the 1:1 and 2:2 intercrops intercepted significantly less PAR than the within-row intercrop in most cases. These differences were clearest at early pod-set to late pod-fill stages of the legumes, particularly of cowpea. The actual PAR intercepted by the intercrops was comparable to the expected PAR interception, if calculated as the sum of 50% of PAR intercepted by each sole crop (Table 3). However, the actual PAR intercepted by the intercrops was 10–31% smaller in the SGS and 17–33% smaller in the NGS compared with the expected PAR interception by the intercrops based on grain yields and radiation use efficiency (RUE) in the sole crops (Table 3). The crops grown in the HF fields intercepted more PAR than the MF and LF fields *(*P <** 0.001; Fig. 3). Soil fertility did not affect PAR interception at the initial growth stages, but did so from flowering to late pod-fill stages.

**Table 3:** Actual and expected percentage intercepted PAR (%IPAR) by intercrops based on plant densities and radiation use efficiencies (RUE) in sole crops in the southern Guinea savanna (SGS) and northern Guinea savanna (NGS) of northern Ghana.

	SGS			NGS		
Cropping pattern	Actual IPAR (%)	Expected IPAR based on plant densities (%)	Expected IPAR based on RUE in sole crops (%)	Actual IPAR (%)	Expected IPAR based on plant densities (%)	Expected IPAR based on RUE in sole crops (%)
MZ-CP						
Mixed	63	60	94	50	47	80
1 to1	59	60	75	45	47	65
2 to 2	56	60	70	45	47	65
Sole MZ	46			36		
Sole CP	74			58		

MZ-SB						
Mixed	70	69	97	53	52	86
1 to1	67	69	81	50	52	71
2 to 2	66	69	76	49	52	69
Sole MZ	55			41		
Sole SB	83			62		

MZ-GN						
Mixed	62	60	88	47	45	75
1 to1	59	60	76	44	45	61
2 to 2	59	60	76	44	45	64
Sole MZ	55			40		
Sole GN	66			51		

CP − cowpea; SB − soybean; GN − groundnut; MZ − maize.

F pr for actual vs expected IPAR based on plant densities:

SGS: MZ-CP: *P* = 0.676; MZ-SB: *P* = 0.235; MZ-GN: *P* = 0.720.

NGS: MZ-CP: *P* = 0.720; MZ-SB: *P* = 0.506; MZ-GN: *P* = 0.886.

F pr for actual vs expected IPAR based on RUE in sole crops:

SGS: MZ-CP (*P *< 0.001, SED = 2); MZ-SB (*P *< 0.001, SED = 2); MZ-GN (*P *< 0.001, SED = 2).

NGS: MZ-CP (*P *< 0.001, SED = 2); MZ-SB (*P *< 0.001, SED = 2); MZ-GN (*P *< 0.001, SED = 1).

Legume biomass yields at mid-pod fill were greater in the SGS than in the NGS (*P <* 0.01) that received less rainfall during the growing season and had soils poorer in fertility (Table 4). Sole legumes had greater above-ground biomass yields than the associated intercrops at both sites (*P <* 0.001; Table 4). However, intercrop biomass yields were larger compared with 50% of the sole legume yields (which corresponds to yields from the same size of land and density as that of the intercrops) with the differences generally being significant only for the within-row intercrops. Cowpea and soybean biomass yields were significantly greater in within-row systems than in distinct rows intercrops, while those of groundnut were comparable. Biomass yields declined with decreasing soil fertility status, but this decline varied among legume species and sites (Table 4). In the SGS, only cowpea and soybean gave larger biomass in the HF field than in the LF field (cowpea: *P <* 0.001; soybean: *P* = 0.016) due to smaller differences soil N and P between the fields, which accounted for smaller variation in the biomass yield compared with variation attributable to planting arrangement (Table 2, Table 5). On the contrary, all the three legume species produced greater biomass in the HF field than in the LF field in the NGS (*P <* 0.001) as the larger differences in soil N and P status between the fields (Table 2) accounted for larger variation in biomass yields relative to variation due to planting arrangement (Table 5). Except for cowpea in the NGS, the legumes produced more biomass (*P <* 0.001) in the second season than in the first season at both sites.

**Table 4:** Above-ground dry matter yield (t ha^−1^) of legumes at mid-pod-fill stage as affected by cropping pattern and fertility status averaged for 2013 and 2014 seasons in the southern Guinea savanna (SGS) and northern Guinea savanna (NGS) of northern Ghana. The SED shows the standard error of difference between means.

Cropping pattern	SGS				NGS			
	HF	MF	LF	Mean	HF	MF	LF	Mean
MZ-CP within row	1.92	1.71	1.30	1.65	1.92	1.73	1.01	1.56
MZ-CP 1:1 rows	1.51	1.32	1.01	1.28	1.45	1.23	0.85	1.17
MZ-CP 2:2 rows	1.27	1.13	0.97	1.12	1.41	1.20	0.73	1.11
Sole cowpea	2.84	2.52	1.37	2.24	2.73	1.82	1.15	1.90
Mean	1.89	1.67	1.16	1.57	1.88	1.49	0.94	1.44
SED (arrangement)				0.09				0.09
SED (fertility)				0.07				0.11
SED (interaction)				0.15				0.17
MZ-SB within row	3.45	3.42	2.96	3.27	3.51	2.09	1.26	2.28
MZ-SB 1:1 rows	3.17	2.77	2.76	2.90	2.37	1.51	0.88	1.59
MZ-SB 2:2 rows	2.97	2.77	2.65	2.80	2.35	1.39	0.92	1.55
Sole soybean	5.84	6.10	5.60	5.85	4.90	2.67	1.69	3.09
Mean	3.86	3.76	3.49	3.70	3.28	1.92	1.19	2.13
SED (arrangement)				0.19				0.14
SED (fertility)				n.s.				0.21
SED (interaction)				n.s.				0.29
MZ-GN within row	1.17	0.86	0.89	0.97	0.87	0.81	0.66	0.78
MZ-GN 1:1 rows	0.86	0.88	0.79	0.84	0.76	0.68	0.56	0.66
MZ-GN 2:2 rows	0.94	0.87	0.87	0.90	0.76	0.69	0.60	0.69
Sole groundnut	1.79	1.74	1.52	1.68	1.40	1.17	0.87	1.14
Mean	1.19	1.09	1.02	1.10	0.94	0.84	0.67	0.82
SED (arrangement)				0.11				0.05
SED (fertility)				n.s				0.05
SED (interaction)				n.s.				0.08

CP − cowpea; SB − soybean; GN − groundnut; MZ − maize.

**Table 5:** Sum of squares, mean squares and F statistics from Analysis of Covariance indicating the sources of variation in above-ground dry matter yield of grain legumes under different spatial arrangement and selected measured soil properties in the southern Guinea savanna (SGS) and northern Guinea savanna (NGS) of northern Ghana.

	SGS					NGS				
Source of variation	d.f.	s.s.	m.s.	v.r.	F pr.	d.f.	s.s.	m.s.	v.r.	F pr.
Block stratum	*Cowpea*									
Covariates	2	4.44	2.22	65.00	<0.001	2	7.18	3.59	39.75	<0.001
Total N	1	0.22	0.22	6.55	0.031	1	6.16	6.16	68.20	<0.001
Avail. P	1	4.22	4.22	123.44	<0.001	1	1.02	1.02	11.30	0.008
Residual	9	0.31	0.03	0.36		9	0.81	0.09	1.06	
Block.*Units* stratum										
Arrangement	3	8.95	2.98	31.02	<0.001	3	4.86	1.62	19.00	<0.001
Residual	33	3.17	0.10			33	2.81	0.09		
Total	47	16.87				47	15.67			

Block stratum	*Soybean*									
Covariates	2	1.15	0.57	1.72	0.233	2	36.02	18.01	53.05	<0.001
Total N	1	0.08	0.08	0.25	0.628	1	35.61	35.61	104.88	<0.001
Avail. P	1	1.06	1.06	3.19	0.108	1	0.41	0.41	1.21	0.299
Residual	9	2.99	0.33	1.68		9	3.06	0.34	1.46	
Block.*Units* stratum										
Arrangement	3	75.00	25.00	126.07	<0.001	3	18.81	6.27	27.03	<0.001
Residual	33	6.54	0.20			33	7.65	0.23		
Total	47	85.69				47	65.54			

Block stratum	*Groundnut*									
Covariates	2	0.24	0.12	3.34	0.082	2	0.59	0.30	18.22	< 0.001
Total N	1	0.00	0.00	0.05	0.821	1	0.50	0.50	30.86	< 0.001
Avail. P	1	0.24	0.24	6.62	0.03	1	0.09	0.09	5.58	0.042
Residual	9	0.32	0.04	0.56		9	0.15	0.02	1.03	
Block.*Units* stratum										
Arrangement	3	5.57	1.86	29.45	<0.001	3	1.79	0.60	37.78	< 0.001
Residual	33	2.08	0.06			33	0.52	0.02		
Total	47	8.21				47	3.05			

**Fig. 4:**
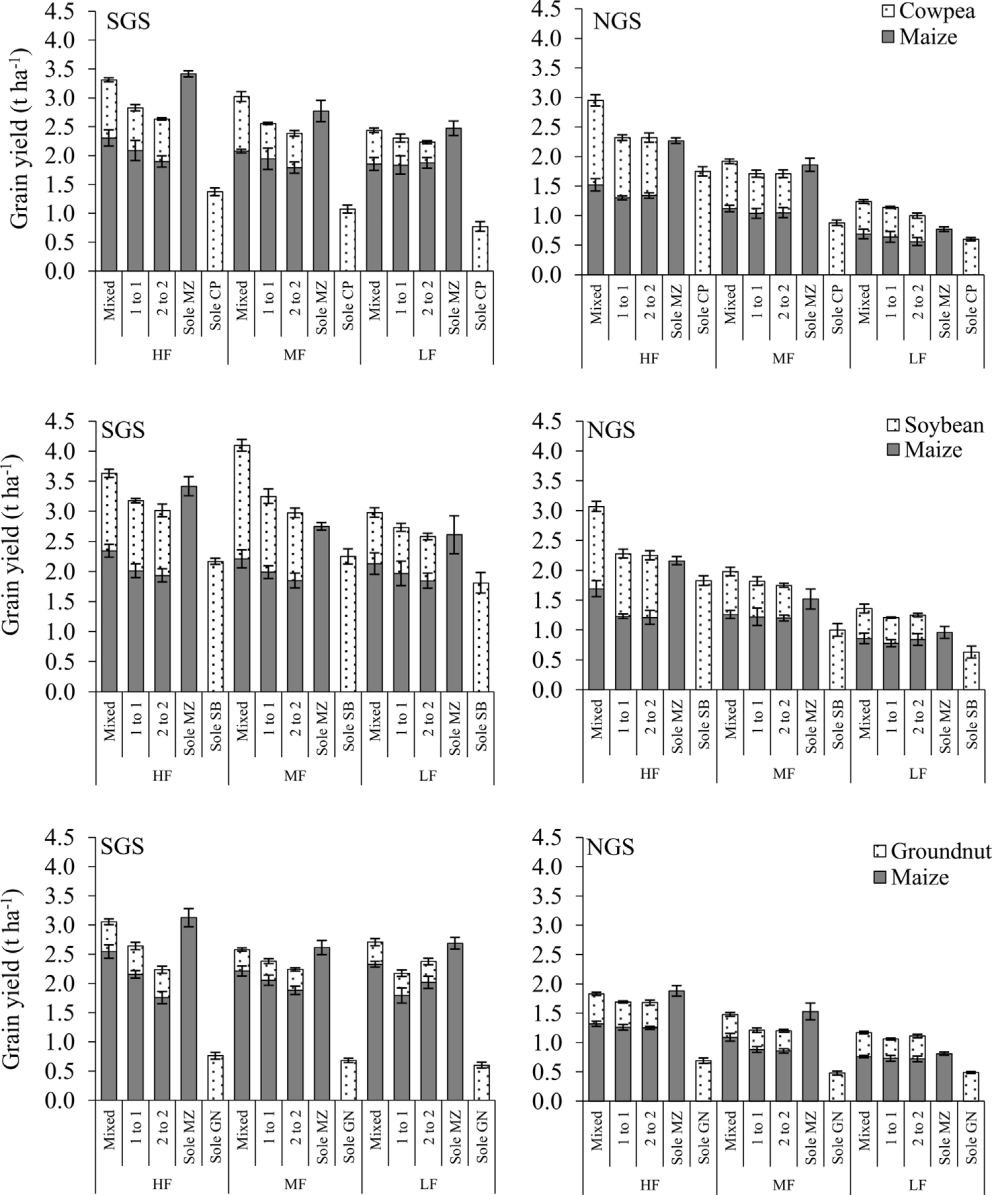
Combined maize and legume intercrop and sole crop grain yields as affected by spatial plant arrangement and soil fertility level, average of 2013 and 2014 seasons in the SGS and NGS of northern Ghana. Error bars represent the standard error of means.

Cowpea grain yield was greater in the NGS than in the SGS (*P* = 0.008), while maize, soybean and groundnut yields were greater in the SGS (*P <* 0.01). Sole crops produced greater grain yields than intercrops at both sites (*P <* 0.001; Fig. 4). Intercropped maize and legume grain yields were larger compared with 50% of the associated sole yields in most cases (*P <* 0.001; Fig. 4). The within-row intercrop pattern in general provided larger maize and legume grain yields than the 1:1 and 2:2 distinct row patterns whereas the latter two had comparable yields. Grain yields differed with cropping season (data not shown). For instance, groundnut produced more grain yield in the second season at both sites (*P <* 0.001). Cowpea grain yield was not significantly affected by season (though the yields declined at both sites in the second season) while soybean grain yield declined in the second season at both sites but significant (*P <* 0.001) only in the NGS. The impact of season on maize grain yield was significant in all maize-legume systems in the NGS while in the SGS, the seasonal effect was significant only for the maize-groundnut system with more maize grain produced in the second season in each case. Combined intercrop grain yields (legume + maize yield) differed between the intercrop patterns only in the HF and MF fields. Grain yields declined with decreasing soil fertility at both sites (*P <* 0.001). This was more evident in the NGS where the differences in soil fertility between fields, (e.g. soil N and P) were larger between the fertile and poorly fertile fields and accounted for much of the observed variation in grain yields compared with that of SGS (Table 2, Table 6). Consequently, the clearer differences in soil fertility status between the fields in NGS were well reflected by grain yields, whereas the decrease in yields with poorer soil fertility was not as clear in the SGS (Fig. 4). The grain yields of sole maize were generally comparable or larger than the combined intercrop grain yields in the HF or MF fields.

**Table 6:** Sum of squares, mean squares and F statistics from Analysis of Covariance indicating the sources of variation in grain yields of legumes and maize under different spatial arrangement and selected measured soil properties in the southern Guinea savanna (SGS) and northern Guinea savanna (NGS) of northern Ghana.

	SGS					NGS				
Source of variation	d.f.	s.s.	m.s.	v.r.	F pr.	d.f.	s.s.	m.s.	v.r.	F pr.
Block stratum	*Cowpea*									
Covariates	2	1.43	0.71	40.5	<0.001	2	5.03	2.51	178.08	<0.001
Total N	1	0.03	0.03	1.58	0.24	1	5.02	5.02	355.36	<0.001
Avail. P	1	1.40	1.40	79.41	<0.001	1	0.01	0.01	0.81	0.393
Residual	9	0.16	0.02	1.3		9	0.13	0.01	0.48	
Block.*Units* stratum										
Arrangement	3	2.01	0.67	49.33	<0.001	3	1.15	0.38	13.1	<0.001
Residual	33	0.45	0.01			33	0.97	0.03		
Total	47	4.04				47	7.28			

Block stratum	*Soybean*									
Covariates	2	2.88	1.44	25.19	<0.001	2	5.92	2.96	97.57	<0.001
Total N	1	1.69	1.69	29.58	<0.001	1	5.92	5.92	195.06	<0.001
Avail. P	1	1.19	1.19	20.81	0.001	1	0.00	0.00	0.09	0.773
Residual	9	0.51	0.06	1.31		9	0.27	0.03	1.05	
Block.*Units* stratum										
Arrangement	3	8.92	2.97	68.07	<0.001	3	1.79	0.60	20.67	<0.001
Residual	33	1.44	0.04			33	0.95	0.03		
Total	47	13.75				47	8.94			

Block stratum	*Groundnut*									
Covariates	2	0.17	0.09	12.07	0.003	2	0.16	0.08	19.95	< 0.001
Total N	1	0.04	0.034	5.52	0.043	1	0.14	0.14	34.72	< 0.001
Avail. P	1	0.13	0.13	18.63	0.002	1	0.02	0.02	5.17	0.049
Residual	9	0.06	0.01	1.93		9	0.04	0.00	1.05	
Block.*Units* stratum										
Arrangement	3	0.69	0.23	63.15	< 0.001	3	0.26	0.09	22.82	< 0.001
Residual	33	0.12	0.00			33	0.13	0.00		
Total	47	1.05				47	0.58			

Block stratum	*Maize*									
Covariates	2	0.79	0.39	7.33	0.013	2	4.86	2.43	92.64	<0.001
Total N	1	0.08	0.08	1.55	0.245	1	4.17	4.17	158.82	<0.001
Avail. P	1	0.71	0.71	13.11	0.006	1	0.69	0.69	26.46	<0.001
Residual	9	0.48	0.05	0.99		9	0.24	0.03	1.12	
Block.*Units* stratum										
Arrangement	3	7.26	2.42	44.45	<0.001	3	2.20	0.73	31.31	<0.001
Residual	33	1.80	0.05			33	0.77	0.02		
Total	47	10.33				47	8.07			

### 3.3 Land equivalent ratios (LER) of intercrops

**Fig. 5:**
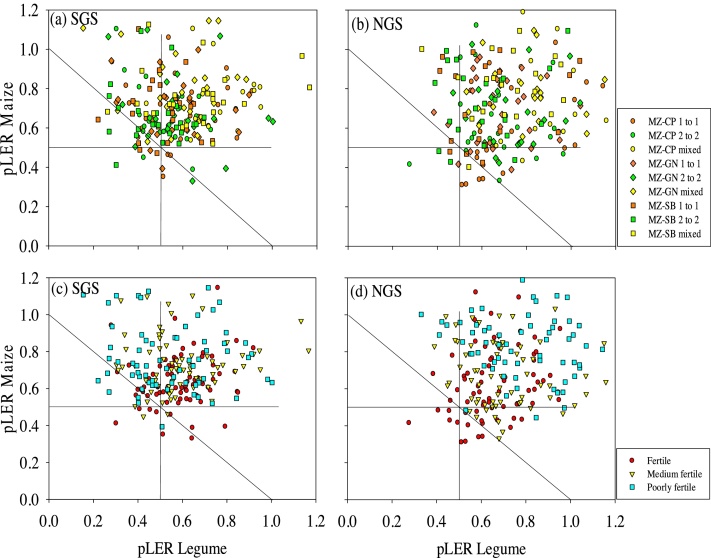
Partial Land Equivalent Ratios (LER) of groundnut, cowpea and soybean intercropped with maize in different spatial planting patterns in (a) the SGS and (b) the NGS, and at different soil fertility levels in (c) the SGS and (d) the NGS for both seasons. MZ-GN refers to maize-groundnut, MZ-CP to maize-cowpea and MZ-SB to maize-soybean intercropping systems. The mixed intercrop refers to the within row intercropping of maize and legume. Data points are from each replicate plot.

**Fig. 6:**
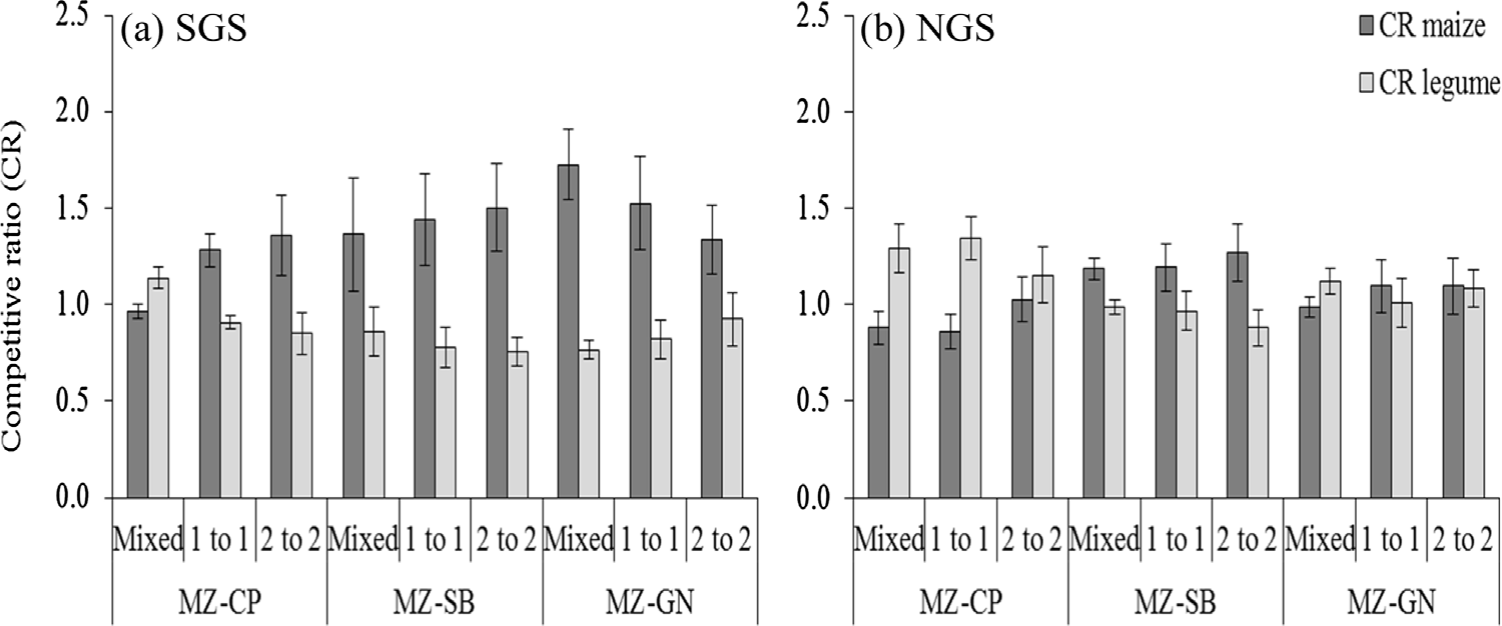
Mean Competitive Ratios (CR) of cowpea, soybean and groundnut intercropped with maize in different spatial arrangements in (a) the southern Guinea savanna (SGS) and (b) the northern Guinea savanna (NGS) of northern Ghana. MZ-GN refers to maize-groundnut, MZ-CP to maize-cowpea and MZ-SB to maize-soybean intercropping systems. The mixed intercrop refers to the within-row intercropping of maize and legume. The error bars indicate the standard error of means.

Mean LER for the different intercrop patterns were all greater than unity which suggested that intercropping led to a more productive use of land than sole cropping (Table 7). Partial LER values of maize were mostly above 0.5 at both sites (Fig. 5). Intercropped maize was more competitive than the legumes, particularly soybean and groundnut in the SGS (Fig. 6). The intercropped legumes performed relatively better in the NGS indicated by more partial LER values above 0.5 (Fig. 5a and b) and reduced competitiveness of maize (Fig. 6a and b) compared with the SGS, especially in intercropping systems with soybean. This led to a 14% greater mean total LER in the NGS than in the SGS (*P <* 0.001; Table 7), with season having no significant effect on the total LER (a mean increase of 2% in SGS and a decline of 5% in the NGS in the second season compared with the first season). The impact of legume species on LER was significant (*P <* 0.046) in the SGS with cowpea-maize and groundnut-maize systems giving larger total LER values than soybean-maize systems (Table 7). This suggests that soybean is less suitable for intercropping than groundnut and cowpea. LER values were greater (*P <* 0.05, generally) in the within-row pattern than in the 1:1 and 2:2 distinct row patterns at both sites.

**Table 7:** Total Land Equivalent Ratios (LER) of maize intercropped with cowpea, soybean and groundnut in different spatial arrangements and at different soil fertility status, averaged over both seasons in the southern Guinea savanna (SGS) and northern Guinea savanna (NGS) of northern Ghana. SED indicates the combined standard error of difference between means.

Cropping pattern	SGS				NGS			
	HF	MF	LF	Mean	HF	MF	LF	Mean
MZ-CP mixed	1.41	1.65	1.54	1.53	1.52	1.51	1.81	1.61
MZ-CP 1:1	1.15	1.28	1.37	1.27	1.18	1.33	1.68	1.40
MZ-CP 2:2	1.10	1.21	1.24	1.18	1.18	1.33	1.47	1.33
Mean	1.22	1.38	1.38	1.33	1.29	1.39	1.65	1.44

MZ-SB mixed	1.28	1.65	1.35	1.43	1.54	1.60	1.80	1.65
MZ-SB 1:1	1.13	1.29	1.21	1.21	1.15	1.43	1.58	1.39
MZ-SB 2:2	1.07	1.18	1.16	1.14	1.13	1.37	1.57	1.36
Mean	1.16	1.37	1.24	1.26	1.27	1.46	1.65	1.46

MZ-GN mixed	1.49	1.40	1.53	1.47	1.45	1.55	1.78	1.59
MZ-GN 1:1	1.34	1.28	1.30	1.31	1.30	1.26	1.58	1.38
MZ-GN 2:2	1.20	1.26	1.40	1.29	1.29	1.28	1.69	1.42
Mean	1.34	1.31	1.41	1.35	1.35	1.36	1.68	1.46
SED (arrangement)				0.03				0.04
SED (fertility)				n.s.				0.05

CP − cowpea; SB − soybean; GN − groundnut; MZ − maize.

Low soil fertility enhanced the performance of the intercropped legumes indicated by larger partial LER values of the legumes in the LF fields compared with partial LER values of legumes in the HF fields (Fig. 5c, d). This effect was more visible in the NGS (Fig. 5d) where the differences in soil fertility parameters (especially N and P) between the HF and the LF fields were stronger than in the SGS (Table 2) and seen in soil N and P being responsible for much of the observed variability in total LER in the NGS than in the SGS (Table 8). This led to greater total LER in the LF fields and the LER values declined with increasing soil fertility status with the values in most cases being smaller (*P <* 0.05) in HF fields (Table 7).

**Table 8:** Sum of squares, mean squares and F statistics from Analysis of Covariance indicating the sources of variation in total Land Equivalent Ratios (LER) of maize-grain legume intercrops under different spatial arrangement and selected measured soil properties in the southern Guinea savanna (SGS) and northern Guinea savanna (NGS) of northern Ghana.

	SGS					NGS				
Source of variation	d.f.	s.s.	m.s.	v.r.	F pr.	d.f.	s.s.	m.s.	v.r.	F pr.
Block stratum										
Legume species	2	0.20	0.10	1.61	0.216	2	0.01	0.00	0.08	0.924
Covariates	2	0.28	0.14	2.26	0.121	2	2.44	1.22	21.53	<0.001
Total N	1	0.28	0.28	4.50	0.042	1	1.82	1.82	32.08	<0.001
Avail. P	1	0.00	0.00	0.03	0.874	1	0.62	0.62	10.97	0.002
Residual	31	1.90	0.06	3.74		31	1.76	0.06	2.43	
Block.*Units* stratum										
Arrangement	2	1.52	0.76	46.36	<0.001	2	1.40	0.70	29.98	<0.001
Arrangement.Legume species	4	0.08	0.02	1.27	0.291	4	0.07	0.02	0.70	0.596
Residual	66	1.08	0.02			66	1.54	0.02		
Total	107	5.05				107	7.23			

### 3.4 Economic profitability of cropping patterns

**Fig. 7:**
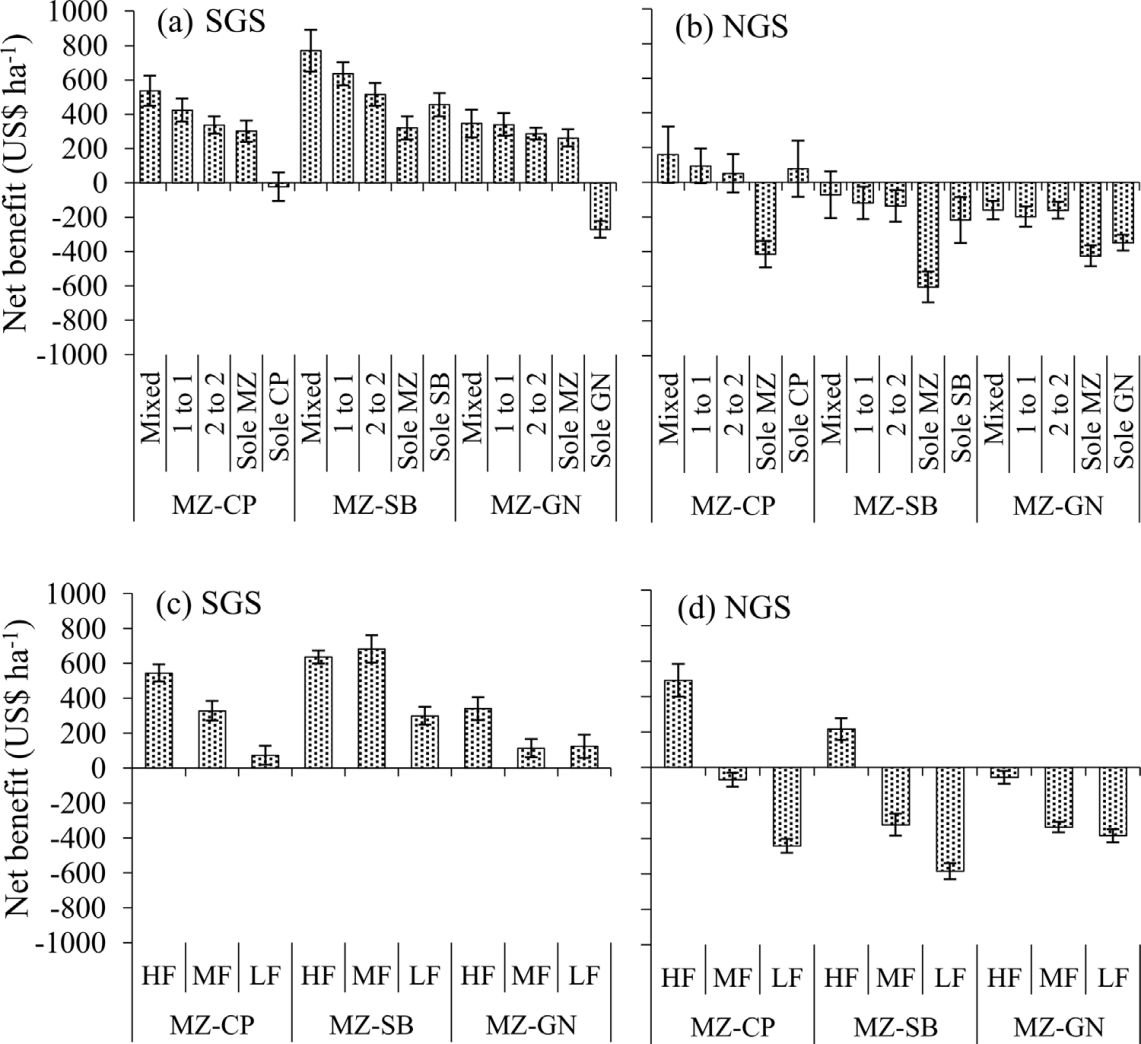
Net benefits from a partial budgeting analysis as influenced by different cropping patterns, in (a) the SGS and (b) the NGS, and as affected by soil fertility status in (c) the SGS and (d) the NGS of northern Ghana. Data presented are averages for two seasons. The error bars indicate the standard error of mean.

The crops in the SGS provided greater economic returns than in the NGS (Fig. 7). The lower net benefits in the NGS resulted from generally poor grain yields and slightly larger costs of production due to higher cost associated with the use of tractor for ploughing. In general, sole legumes were more profitable than sole maize except sole cowpea and groundnut in the SGS due to relatively low grain yields. High labour requirements to produce legumes (Table 1b) contributed to the smaller returns of sole legumes. The extra time needed for sowing, urea application to maize and weeding in the within-row intercrop system led to consistently greater TC than in the other cropping patterns (data not shown). The distinct row intercrops had larger TC than sole maize due to higher labour costs of legume cultivation. The TC of the distinct row intercrops was smaller than sole legumes which also had larger TC than sole maize (data not shown) due to higher labour requirements for legumes production (Table 1b). However, the greater grain yield in intercropping resulted in larger net benefits than in sole cropping of maize and legumes (*P <* 0.001), with the benefits generally larger with the within-row intercrops (Fig. 7a, b). The larger grain yields obtained by growing crops in the HF fields led to significantly (*P <* 0.001) greater net benefits, which declined with decreasing soil fertility (Fig. 7c, d).

## 4 Discussion

### 4.1 Biophysical characteristics and crop production

Soil nutrient concentrations were generally low at both sites (Table 2) in relation to critical values for sub-Saharan Africa (Fairhurst, 2012), and are representative for farmers’ conditions in northern Ghana (Buri et al., 2010). The differences in soil fertility characteristics between the two sites may be attributable to differences in soil types, as well as past farmers’ management. For example, crop residues were commonly retained in the field in the SGS, while they were often exported in the NGS to feed livestock or to be composted. The greater biomass and grain yields produced in the SGS compared with the NGS (Table 4; Fig. 4) were consistent with the differences in rainfall and soil fertility characteristics that were more favourable for crop growth in the SGS. The lower amount of rainfall received in the NGS (Fig. 1) and the predominantly sandy soils (Table 2) probably led to less water availability in the NGS, also contributing to smaller yields.

### 4.2 Cropping pattern and soil fertility effects on grain yields

The comparable maize grain yields in intercrops and sole maize in the LF field in NGS that had low soil N status corroborates the finding of Ahmed and Rao (1982) who also observed comparable yields for intercropped and sole maize grain under low soil N conditions. This might be because under low N conditions, there will be less competition for radiation between the intercrop components. Also, there could be a more marked impact of N_2_ fixation of the intercropped legume on the maize component under low soil N conditions than under high soil N status. The greater grain yield of maize and legumes (both intercrops and sole crops) in the fertile fields compared with the poorly fertile fields mirrored the soil fertility gradient between the fields at both sites (Table 2; Fig. 4). In particular, the grain yield differences induced by the soil fertility gradient was remarkably consistent in the NGS where stronger differences in soil fertility between the fields were observed (Table 2; Fig. 4). Our results agree with the findings of other authors such as Oikeh et al. (1998) in the Guinea savanna of Nigeria and Ojiem et al. (2014) in Western Kenya who observed a consistent decrease in maize and legume grain yields in response to decreases in soil fertility among fields.

### 4.3 Resource use and intercrop productivity

LER values greater than one for the intercrop patterns (Table 7) indicate a more efficient and productive land utilization by intercrops compared with the sole crops (Willey, 1985). However, except for maize and soybean intercrops, the combined intercrop grain yields (maize + cowpea or groundnut) were smaller than that of sole maize, particularly in the HF and MF fields. This may be a disincentive for farmers in terms of meeting household food needs if maize is prioritised above the legumes. Given that our trial had a replacement intercrop design, testing of additive intercrops would be worth testing. While the amount of PAR intercepted by the intercrops was comparable with that of 50% of each sole crop (Table 3), the combined intercrop grain yields were 26–43% larger than the sum of 50% of each sole crop yield. This suggests that RUE in intercrops was greater than in sole crops, as also observed in earlier studies (Reddy and Willey, 1981, Marshall and Willey, 1983, Willey, 1990, Keating and Carberry, 1993). Other authors (Awal et al., 2006, Gao et al., 2010) reported that intercropped maize and sole maize had comparable radiation extinction coefficients and radiation use efficiencies. By contrast, intercropped legumes, for example groundnut, had smaller extinction coefficients and greater radiation use efficiencies than sole groundnut (Harris et al., 1987, Keating and Carberry, 1993, Awal et al., 2006, Gao et al., 2010). The legumes in intercrops fixed 15–97 kg ha^−1^ of N_2_ representing 67–71% of N_2_ fixed by respective sole crop legumes (Kermah et al. submitted), and this may have improved soil N availability to maize in intercrops in the second season, relative to sole maize. Improved leaf N content and photosynthetic activity of maize in intercrops may have led to enhanced RUE (Sinclair and Horie, 1989, Gimenez et al., 1994) of the intercropped maize in the second season.

We did not rotate the sole legume and sole maize treatments in the second season. With crop rotation, the maize would have benefitted from residual N of the legume from the first season. Rotating sole legume and sole maize could lead to avoidance of pests and diseases build-up relative to continuous intercropping of maize and legumes (Stevenson and Van Kessel, 1996). Intercropping, however, can result in better suppression of pests and diseases than continuous cropping of either crop alone (Trenbath, 1993). The different cropping sequences of the different fields could have led to differences in build-up of soil borne pathogens and insect pests that would confound the effect of soil fertility status on crop performance. However, we did not observe differences in pest and disease attack among the crops grown in the different fields which suggests that such effects were not important during the study.

The within row intercrop was more productive than the other intercrop planting patterns, as previously reported in Central Mozambique (Rusinamhodzi et al., 2012). This suggests that the current recommendations of a distinct row intercrop pattern involving two rows of maize alternated with four rows of cowpea that is promoted in the NGS of Nigeria (Ajeigbe et al., 2010) needs to be revisited. However, the distinct row intercrop design is more amenable to mechanisation of some activities, such as sowing, weeding, fertiliser application, though these activities are currently performed manually by smallholders in the region. The larger productivity of the within row system may have been the outcome of a slightly better radiation capture (Fig. 2) coupled with an efficient use of the intercepted PAR resulting from the differing canopy architecture compared to distinct row systems (Reddy and Willey, 1981).

Our results show that in maize-legume intercropping, the maize is more competitive than the legume under high soil N conditions as in the SGS leading to a relatively small contribution of legume (Table 2; Fig. 6a) to the total LER (Table 7). Under low soil N conditions such as in the NGS, the competitiveness of the maize is reduced and the intercropped legume gains in relative competitiveness (Table 2; Fig. 6b; Chang and Shibles, 1985, Midmore, 1993). This is largely due to the ability of legumes to fix N_2_ (Kermah et al., submitted) and the apparent reduced competition for radiation between the intercrop components in poorly fertile fields leading to reduced shading of the legume by the intercropped maize crop. This resulted in a competitive balance (similar competitiveness and contributions of the intercrop components to the total LER (Yu et al., 2016)) between the maize and legume intercrop components in the NGS (Fig. 5d; Fig. 6b) resulting in a greater total LER, particularly in the LF field (Table 7; Ofori and Stern, 1986, Yu et al., 2016). Soils in the NGS had a poorer N status (Table 2) suggesting that LERs increase with decreasing levels of soil N, as reported by other studies (Searle et al., 1981, Ahmed and Rao, 1982, Ofori and Stern, 1986). Greater LERs in the poorly fertile fields, and in general in the NGS with more marginal growing conditions than in SGS (Fig. 1; Table 2, Table 4) indicate that intercropping is more advantageous under low soil fertility conditions.

### 4.4 Economic profitability as affected by cropping pattern and soil fertility

Greater grain yields in the within-row intercrop systems led to larger net benefits than distinct row intercropping systems, despite the higher labour input in within-row systems. The lower net benefits of sole maize in the NGS (Fig. 7b) were the outcome of relatively poor grain yields, making sole cropping of maize economically less attractive in the NGS and farmers may be better off intercropping maize with grain legumes, especially cowpea. Crop production in HF fields was more profitable than in MF and LF fields due to larger grain yields (Fig. 4; Fig. 7). This indicates that poor soil fertility leads to smaller net benefits, as reported for maize-legume intercrops in Western Kenya by Ojiem et al. (2014). This is a common feature in the Guinea savanna agro-ecology where smallholder farmers are trapped in a vicious cycle of poor soils leading to poor grain yields and consequently poor economic benefits.

## 5 Conclusions

The observed advantage of intercrops over sole crops was associated with an enhanced radiation use efficiency (RUE) by intercrops. While legumes may have achieved a higher RUE in intercropping systems due to their ability to perform relatively well under low-radiation conditions, maize in intercropping may have had a higher RUE due to improved soil N availability in the second season. Intercropping of maize and grain legumes within the same row appears the most productive and lucrative pattern that can be exploited by farmers in the Guinea savanna, though distinct row intercrop patterns are also generally more profitable than sole crops. Benefits of maize-legume intercropping are greater under low soil fertility conditions, presumably due to reduced competition for light and possibly enhanced benefits from legumes’ ability to fix N_2_. Nevertheless, overall cropping is more profitable in fertile fields due to larger absolute grain yields. Our results show a good potential for maize-legume intercropping for farmers in the Guinea savanna, particularly under more marginal conditions.
